# The Bidirectional Relationship between Metabolism and Immune Responses

**DOI:** 10.15190/d.2013.6

**Published:** 2013-12-31

**Authors:** Forum M. Raval, Barbara S. Nikolajczyk

**Affiliations:** Boston University School of Medicine, Department of Microbiology, Boston, MA, USA

**Keywords:** Immunometabolism, obesity, type 2 diabetes, immune cell activation, nutrients, diet-induced obesity

## Abstract

Immunometabolism investigates the multiple links between the immune system and metabolism. One main focus of immunometabolism investigates how obesity impacts the immune system and pro-inflammatory immune cell function, leading to metabolic diseases, including type 2 diabetes (T2D). The second focus stresses the metabolic changes that dictate immune cell activation. Several groups have studied these two arms of the field individually, but work that integrates both topics will be required to develop an accurate understanding of how immune cells and metabolic pathways collaborate in obesity and obesity-associated T2D. Investigations of the relationships among obesity-induced changes in the nutritional environment, immune cell activation, and immune cell metabolism may lead to novel and efficacious therapies for obesity-associated disorders such as insulin resistance (IR) and T2D. This review outlines recent insights into two related processes: 1. the role that energy utilization plays in immune responses and 2. the immune cell functions that drive obesity and T2D. Herein, we begin to consider how shifts in available fuel sources in obesity and T2D impact the immune response to both pathogens and chronic over nutrition.

## Role of immune cells in obesity-associated type 2 diabetes (T2D)

**Figure 1:**
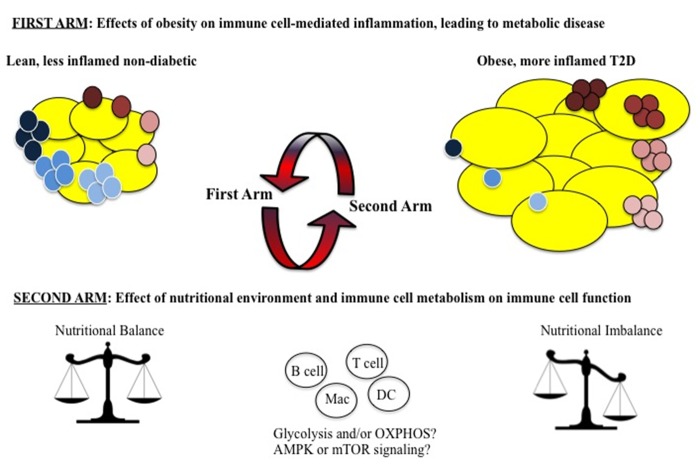
The two arms of immuno-metabolism The first arm investigates how obesity affects the immune system and how it promotes inflammation. The brown-tinted cells represent pro-inflammatory cells such as Th1, Th17, M1 macrophages and (under the conditions of obesity) B cells. The blue-tinted cells represent anti-inflammatory cells such as Th2, M2 macrophages and Tregs. The second arm of immunometabolism investigates how metabolic changes affect immune cell activity. The red arrows suggest that one arm influences the other. Studying both arms of immunometabolism in combination will provide for a better understanding of the roles of the immune system, various fuel sources, and metabolic pathways in obesity and the associated metabolic disease.

The goal of the relatively new field of immunometabolism is to understand the relationship between the immune system and metabolism. Two major arms of the field have emerged (**Figure 1**). The first arm of immunometabolism investigates how obesity affects the immune system and promotes the inflammation that leads to pancreatic islet failure and/or insulin resistance (IR). Studies of these obesity-triggered processes justify tests of therapies that target the immune system in attempts to alleviate both metabolic disease and its inflammation-associated co-morbidities, such as cardiovascular disease^1^. The second arm of immunometabolism queries the immune cell-intrinsic metabolic changes needed to fuel immune system activation. Although both subfields of immunometabolism have been advancing at break-neck speed, insights into these processes beg the unanswered question of how immune cell activation changes along with the balance of fuel sources during obesity and obesity-associated metabolic disease, such as T2D.

Most information on the role immune cells play in T2D has been generated by studies on adipose tissue or blood, and data from our lab and others suggest that at least some (though not all) immune cell characteristics/functions are similar in immune cells from the two tissue types^2,3^. Arguably the best understood immune cell in obese adipose tissue is the macrophage. “M2” macrophages, which play important roles in tissue repair and are considered anti-inflammatory, dominate lean adipose tissue, while interferon gamma-secreting “M1” macrophages, widely considered pro-inflammatory, are the predominant immune cell type in obese/expanding adipose tissue^4^. The (perhaps overly simplified) transition from an M2- to M1-dominated fat pad is likely due to blood immune cells that infiltrate adipose tissue in response to over-nutrition, then respond to adipocyte-generated adipokines and fatty acids (FAs) that locally promote M1 differentiation. This model of the adipose tissue environment as the major determinant of immune cell function has been developed from studies that showed monocytes from lean mice preferentially differentiate into pro-inflammatory M1 macrophages in adipose tissue of obese/IR mice, while monocytes from obese/IR mice do not maintain inflammatory characteristics after transplantation into lean mice^5^. Interestingly, obesity also reduces the iron content of adipose tissue macrophages (ATMs)^6^. This finding suggests that the effect of FAs on ATM iron metabolism may contribute to the phenotypic cellular changes that, in combination with adipose tissue recruitment, culminate in obesity-associated inflammation. It is not known whether lymphocytes are similarly dominated by the environment, although cell-intrinsic changes have been identified in B and T cells from T2D patients and obese/IR mice^3,7-9^. Remarkably, despite the fact that adipose tissue contains lymphocytes and myeloid-derived immune cells, it does not architecturally resemble a lymphoid organ. Rather, the immune cells aggregate in small, disorganized inter-adipocyte clusters in adipose tissue, isolated as the stromal vascular fraction (SVF) of adipose tissue preparations^4,10^.

In the case of macrophages, adipose-associated immune cells can also extend long processes that engulf dying adipocytes in so-called “crown-like” structures that may or may not contain additional immune cell types^7,11^.

Although much of the focus of immune cell roles in obesity/IR has been on macrophages for the past decade, T cells and B cells in the adipose tissue SVF are increasingly recognized as major sources of obesity-associated inflammation. Initial studies identified a correlation between body mass index and CD3 mRNA, which encodes one polypeptide of the T cell receptor^7,12^. These data, among other early pieces of work, suggested that CD8^+^ and/or CD4^+^ T cell subsets play a direct role in regulating the inflammatory environment, and furthermore control adipose tissue macrophages^4^. Definitive studies on CD8^+^ T cells showed that depletion of these cells in mice reduced adipose tissue macrophage infiltration, decreased adipose tissue inflammation and ameliorated IR^13^. CD8^+^ T cells are also increased in adipose tissue of obese humans, although a likely pro-inflammatory function has not been reported^14^. In contrast to the relatively simple pro-inflammatory role of CD8^+^ T cells in obesity, the contribution of CD4^+^ T cells to obesity-associated inflammation and IR is complex, because CD4^+^ T cells can differentiate into distinct effector T cell populations with opposing functions^15^. CD4^+^ Th1 and Th17 cells secrete pro-inflammatory cytokines like IFN-γ and IL-17A that correlate with IR in adipose tissue from obese patients, thus are considered pro-IR^1,16^. In contrast, regulatory CD4^+^ T cells (Tregs) naturally inhibit inflammatory Th1 and Th17-driven responses. The widespread decrease in Treg percentages and numbers in obesity indicates that loss of Tregs likely reinforces the expansion of the inflammatory CD4^+^ T cell subsets that play vital roles in obesity-associated IR^2,7^.

Studies from our lab and others also indicate the importance of B cells in inflammation in obesity and T2D. Multiple labs have shown that B cell-null New Zealand Obese (NZO), µMT and B cell-depleted mice are protected from IR in response to obesity^2,9,17^. Our complimentary studies on B cells from T2D patients show that these cells constitutively and inducibly secrete a pro-inflammatory cytokine profile compared to B cells from non-diabetes subjects^8^. This cytokine profile is characterized by extremely low production of IL-10, an anti-inflammatory cytokine shown to prevent insulin resistance^8,18^. Recent work from our lab and others similarly indicated that IL-10 secreting “regulatory” B cells are decreased in murine obesity/IR^2,19^. Furthermore, artificial increases in regulatory B cells may protect against metabolic disease, as indicated by preliminary studies that highlight the clinical promise of regulatory B cells sparked by the seminal characterization work^1,2,19^. B cell antibodies have also been implicated in obesity-associated IR by multiple studies^9,20^. While one study suggested the presence of autoreactive obesogenic antibodies in obese individuals^9^, a second study failed to confirm such autoreactive antibodies associate with obesity^2^. A third study unexpectedly showed that antibodies from obese individuals function to protect ghrelin from enzymatic breakdown, which resulted in a chronic increase in appetite^20^. The identification of this non-immunological role for B cells as unique sources of antibody raises the possibility that novel mechanisms link the immune system to obesity/IR. Finally, B cells may promote obesity-associated inflammation through their supportive role in mediating pro-inflammatory T cell function^3,7,9,21^. An example of this was shown by studies in samples from T2D subjects, wherein human B cells supported Th17-mediated IL-17A production through a contact-dependent mechanism^2^. Although details in the level of responses, specific cytokines, or antibody targets differ somewhat between mice and humans, the significant overlap in data describing the role of B cells in obesity-induced inflammation and diabetes in obese/IR mice and human T2D samples strongly supports the conclusion that B cells play a critical role in T2D.

## Role of metabolism in immune cell activity and maintenance

An immune response requires a large amount of energy that can be generated from a variety of nutrients including carbohydrates (glucose), FAs, and amino acids^22,23^. Nutrients can be used to fuel two major metabolic pathways to generate energy-storing adenosine triphosphate (ATP). In the first pathway, glycolysis converts glucose to pyruvate in the cytoplasm, and phosphates from glycolytic intermediates are transferred to adenosine diphosphate (ADP) to generate ATP. The second pathway, the tricarboxylic acid (TCA) cycle, generates electron donors for the electron transport chain to fuel oxidative phosphorylation (OXPHOS), which generates ATP within the mitochondria. Acetyl-CoA, derived from the glycolytic end product pyruvate, can enter the TCA cycle. Other nutrients such as glutamine and FAs can be similarly metabolized to acetyl-CoA and enter the TCA cycle to drive OXPHOS. Under hypoxic conditions, cells produce ATP only via glycolysis and convert pyruvate into lactate rather than acetyl-CoA. Other times, even in the presence of sufficient oxygen, cells can use glycolysis rather than OXPHOS^23^, a process known as the Warburg effect. Glycolysis in an oxygen-rich milieu drives energy production most notoriously in cancer, but also fuels immune responses^23,24^.

An effective anti-pathogen response requires immune cells to grow, proliferate, develop effector and/or memory functions, or die. These processes require not only a specific molecular program, but also essential nutrient uptake and energy metabolism. Thus immune cells such as T cells, B cells, dendritic cells and macrophages rapidly shift from a resting to a highly metabolically active state in the presence of appropriate co-stimulation and cytokines, characteristic of the natural response to pathogens.

Multiple reports describe the difference in T cell metabolism under conditions of rest, proliferation, effector function (such as cytokine production, B cell help, target killing), and memory generation. Both naive and memory T cells break down glucose, lipids and amino acids via OXPHOS to generate energy to maintain quiescence and survival^22^. Other pathways, such as glycolysis, may be involved in fueling resting and memory T cells, but such roles have not been rigorously established^23^. Growth factors, including cytokines, are required for the uptake of essential OXPHOS nutrients listed above, which promote the survival of resting and memory T cells. For example, cytokines such as IL-4 and IL-7 can increase the expression of nutrient transporters, such as Glut1, on the cell surface. In the absence of appropriate cytokines, glycolysis decreases, thus limiting mitochondrial substrates for OXPHOS and inhibiting both major metabolic pathways^22,25^. In addition to roles in the maintenance of resting and memory T cells, glycolysis and OXPHOS play critical roles during T cell activation. Initial antigen recognition, activation of T cell receptor-associated signaling pathways, and T cell proliferation require multiple mechanisms to activate glycolysis and/or OXPHOS, including co-stimulation, nutrient uptake and cytokine-mediated stimulation^7,23,26^.

Following T cell activation, metabolic pathways play important roles in effector responses of known T cell subsets. To examine the regulation of cellular metabolism during an immune response, and to identify the effects of metabolism on CD8^+^ T cell populations, Pearce and colleagues measured bioenergetic profiles of naïve, effector and memory CD8^+^ T cells from mice infected with *Listeria monocytogenes. *The oxygen consumption rate, a marker for OXPHOS, was higher in resting than effector CD8^+^ T cells. Effector CD8^+^ T cells also had a higher basal extracellular acidification rate, a consequence of lactic acid production, and thus a marker for glycolysis. These studies show that memory CD8^+^ T cells are less glycolytic and depend more on OXPHOS for long-term survival and function^27^.

Metabolic pathways play similarly critical and stage-specific roles in the function of CD4^+^ T cells. Effector CD4^+^ T cells such as Th1, Th2, and Th17 cells express high amounts of the glucose transporter Glut1, and thus are likely highly glycolytic. On the other hand, Tregs, which can suppress T cell responses and maintain homeostasis, express low concentrations of Glut1 and show high lipid oxidation rates^28^. Additionally, activated protein kinase (AMPK) and mammalian target of rapamycin (mTOR) play important and diametrically opposed roles in metabolism and immune responses^22^. AMPK is involved in the regulation of energy level throughout the body, including in T cells, by responding to variations in hormonal and nutritional signals. AMPK is overall catabolic. mTOR, one of the downstream targets of AMPK, is an intracellular nutrient sensor in T cells (among others) that controls cell growth and metabolism thus is characterized overall as anabolic^22,26,29^. The inverse relationship between AMPK and inflammatory gene expression in adipose tissue of obese subjects, and the lower expression of AMPK in adipose tissue from obese/insulin resistant compared to obese/insulin sensitive subjects are consistent with the possibility that AMPK keeps inflammation and metabolic disease at bay in obese individuals^30^. These human analyses complement mouse studies, showing that anti-inflammatory Tregs have more highly activated AMPK, as evidenced by AMPK phosphorylation, compared to effector CD4^+^ T cells^29^. These studies also showed that AMPK activation and mTOR inhibition resulted in increased lipid oxidation to drive Treg function, thus dampen a T cell-mediated immune response^28^. T cell receptor signaling and CD28 co-stimulation also activate AKT^22^ which in turn, increases glucose uptake and glycolysis^31^.

Although metabolic changes in immune cell activation have been most intensively studied in T cells, other immune cells such as dendritic cells and macrophages also require shifts between the two basic metabolic pathways to fuel cellular responses. Resting dendritic cells, like resting T cells, depend on OXPHOS for energy generation, then switch to a glycolytic metabolic program post-activation^32^. Macrophages similarly require glycolysis and/or FA oxidation/OXPHOS to fuel specific cellular states^29^. M1 pro-inflammatory macrophages utilize glycolysis to generate ATP, while M2 macrophages preferentially utilize OXPHOS^33^. Landmark work showed that the metabolite succinate is necessary for macrophages to switch from OXPHOS to glycolysis upon activation and to subsequently secrete pro-inflammatory cytokines^34^. Additionally, AMPK activation supports mitochondrial FA oxidation, which is observed in M2 macrophages that are activated by IL-4^29^.

In depth studies of the role of glycolysis and OXPHOS in the generation of B cell-derived plasma cells, antibody switching/production and the induction of memory/long-lived B cells have not been reported. Nevertheless, it has been shown that crosslinking the B cell antigen receptor results in a rapid increase in glucose uptake/glycolysis in B cells^35^. Furthermore, IL-4, a cytokine required for B cell survival and function, is necessary for B cells to up-regulate Glut1 on the cell surface, which subsequently increases glycolysis^36^. mTOR and AKT signaling also support multiple pathways that are key for B cell survival and growth^37^. Overall, glycolysis/and or OXPHOS, in addition to mTOR/AKT signaling pathways, may play similar roles in B cells, T cells, dendritic cells and macrophages.

## Role of the nutrient environment on immune cell responses and inflammation

The importance of extracellular nutrient uptake and downstream metabolic pathways in immune cell function raises the possibility that shifts in available fuel sources due to obesity and/or obesity-associated T2D change the function of immune cells thus the effectiveness of immune responses. The lipolytic imbalance that characterizes T2D can create an environment with an excess of lipid-derived nutrients that may significantly alter immune cell function. Lipolysis occurs in multiple tissues including adipose tissue, muscle and liver, and leads to broad systemic changes that may impact both circulating and tissue-associated immune cells^26^. In addition to obesity-associated changes in lipolysis, other changes in the nutrient environment may play critical roles in obesity-associated inflammation. An imbalance in nutrients such as FAs may induce inflammatory cytokine production by a variety of cell types. For example, peritoneal macrophages treated with a mixture of the saturated FAs palmitate and oleate secrete higher amounts of IL-6 and TNFα compared to BSA-treated macrophages^38^. Similarly, palmitate activates IL-6 expression in human myotubes and coronary artery endothelial cells^39^. In contrast, unsaturated FAs such as EPA (Eicosapentaenoic acid) or DHA (Docosahexaenoic acid) induce anti-inflammatory signals in skeletal and adipose tissue by activating AMPK and PPARγ, both of which regulate FA storage and glucose metabolism. Unsaturated FAs furthermore inhibit NF-κB, a transcription factor broadly implicated in inflammation and IR^40^. Perhaps surprisingly, pretreatment of macrophages with polyunsaturated FAs did not affect inflammatory cytokine production^38^.

Environmental nutrients also determine T cell function. One group showed CD4^+ ^T cells responded to adipocyte-released FAs by producing increased amounts of pro-inflammatory cytokines such as IFN-γ and IL-17. FAs also increased T cell proliferation. One complication of this analysis was that the subjects who donated the T cells had osteoarthritis, a pro-inflammatory condition that may have had an unquantified effect on T cell responses. Regardless, this study raises the possibility that cell-intrinsic and disease-associated changes may combine with an altered nutrient environment to significantly affect T cell responses^10^.

**Figure 2:**
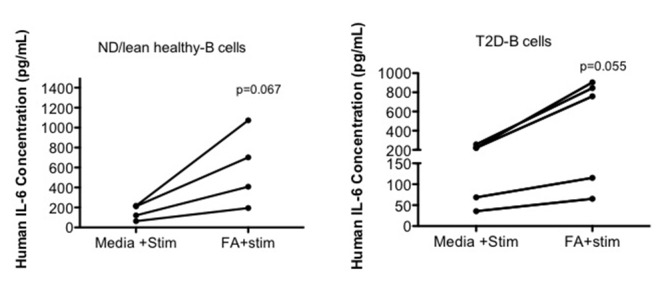
Increase in IL6 production by healthy and T2D B cells upon oleate incubation with cell-specific stimuli B cells from PBMCs of healthy or T2D blood were isolated using a MACS separation B cell kit (negative selection for CD19+ cells; Miltenyi). Oleate was complexed to BSA at 1:4 and incubated with B cells for 20h prior to CpG stimulation. B cells were stimulated with 0.25ug/ml CpG for 40 hours. Supernatant from cultured B cells was used to determine IL-6 production. An ELISA kit for human IL-6 from R&D was used to determine IL-6 concentrations. Subjects were selected as previously described^2^. ND (non-diabetic) *Paired t test confirmed the trend towards significance for CpG stimulated B cell-IL-6 production to be higher in the presence of oleate.

The impact of nutrient environment vs. cell-intrinsic changes of naïve immune cells in an obese/T2D environment have not been reported, despite the importance of understanding factors that influence how quickly naïve cells switch their metabolic programs and become activated. We preliminarily tested the possibility that both disease-associated and cell-intrinsic changes combined with changes in the nutrient environment to determine immune responses in obesity-associated T2D. We treated purified human B cells with saturated FAs (oleate) for 20 hours to model chronic nutrient changes in obesity/T2D, then mimicked pathogen engagement with the B cell stimulant CpG. B cells from either healthy or T2D subjects secreted higher amounts of IL-6 following oleate pre-incubation compared to B cells stimulated with CpG alone (**Figure 2**). These results are consistent with other studies that showed saturated FA, in combination with cell type-specific stimuli, increased pro-inflammatory cytokine production in murine macrophages^38^. To the contrary, mouse T cells responded to a mixture of T cell-specific stimuli plus oleate and palmitate with *decreased* amounts of pro-inflammatory Th1 cytokines compared to responses to T cell-specific stimuli alone^28^. Our work thus far has shown no consistent response of human T cells to FAs, but current data are consistent with the preliminary interpretation that saturated FAs have different effects on T and B cell responses. Regardless, **Figure 2** data suggest that B cell responses are nutrient-dependent, although, unlike IL-10 producing B cells, IL-6 produced by B cells utilizes a disease-independent mechanism, consistent with our previous work^21^. It is probable that multiple factors and nutrients function together in regulating immune cell responses, but combinatorial responses to multiple nutrients have not been tested. Overall, a mixture of nutrient environment and cell-intrinsic changes may shift the metabolic response to promote chronic, low-level inflammation in obesity. The growing number of immunomodulatory (largely anti-inflammatory) treatments for obesity/T2D patients has proven to be modestly effective (at best) for normalizing measure of metabolic health^41-44^. Perhaps understanding the metabolic dynamic of immune cell responses will increase success of strategies that aim to shift immune cell response to decrease chronic diabetogenic inflammation or inflammation-associated comorbidities. Metformin, arguably the most commonly prescribed T2D drug, functions not only to reduce glucose production by the liver, but also to down-regulate inflammation through its ability to activate AMPK^45^.

However, the molecular action of metformin is surprisingly not entirely understood, and metformin is not effective for all T2D patients^45^.

Understanding the “cell-intrinsic” versus “environmental” control of immune cell responses in obesity/T2D may pave the way for targeted therapies that, for example, prevent the environmental dominance of macrophage function^5^, and ameliorate the shift from pro-inflammatory glycolysis to less inflammatory OXPHOS. Understanding how the metabolic environment together with cell-intrinsic changes modifies the immune response will also be critical for treating the sub-optimal immune response that culminates in impaired wound healing and increased susceptibility to at least some pathogens in obesity/T2D^46^. In order for our collective work to be realized in the clinic, we propose that the field must move forward to combine investigations that integrate both arms of immunometabolism (**Figure 1**).

## KEY POINTS

- **Immune cells are major sources of inflammation that promotes obesity-associated insulin resistance**.
- ****Metabolic pathways ****(that depend on environmental nutrients)**** are crucial for immune cell quiescence & activation****, and are also ****key determinantsof**** immune cell-mediated ****inflammation****.
- ****How do obesity-associated changes in the nutrient milieu impact immune cell activation, thus the chronic inflammation that causes insulin resistance in obesity?****
